# Photocatalytic Property of TiO_2_-Vermiculite Composite Nanofibers via Electrospinning

**DOI:** 10.1186/s11671-015-0977-1

**Published:** 2015-07-01

**Authors:** Chao Tang, Meiling Hu, Minghao Fang, Yangai Liu, Xiaowen Wu, Wenjuan Liu, Meng Wang, Zhaohui Huang

**Affiliations:** School of Materials Science and Technology, Beijing Key Laboratory of Materials Utilization of Nonmetallic Minerals and Solid Wastes, National Laboratory of Mineral Materials, China University of Geosciences (Beijing), Beijing, 100083 China

**Keywords:** TiO_2_, Vermiculite, Photocatalysis, Electrospinning

## Abstract

Titanium dioxide (TiO_2_) is one of the most common photocatalysts. In this study, TiO_2_-vermiculite composite nanofibers with a mesh structure and a diameter of approximately 300 nm were prepared via sol–gel approach combined with electrospinning technique. The samples were characterized by X-ray diffraction, scanning electron microscopy, ultraviolet–visible spectroscopy, etc. The photocatalytic property was also evaluated. The TiO_2_-vermiculite composite nanofibers annealed at 550 °C for 3 h exhibited the best absorption and photo-degradation ability for the treatment of methylene blue. The results implied that the combination of mineral vermiculite powders with TiO_2_ enhanced the absorption-degradation performance of the as-prepared photocatalytic materials, consequently promoting the materials’ ability to degrade methylene blue.

**Electronic supplementary material:**

The online version of this article (doi:10.1186/s11671-015-0977-1) contains supplementary material, which is available to authorized users.

## Background

Titanium dioxide (TiO_2_) is one of the most common photocatalytic materials due to its unique energy band structure and non-toxicity. Several methods have been reported to synthesize TiO_2_ nanoparticles, nanowires, nanotubes, nanofibers, etc. However, most of the photocatalytic materials currently used consist of nanoparticles [1]. But the photodegradation rate of photocatalytic materials is rather low because the powders easily agglomerate. Moreover, the band gap of TiO_2_ is 3.2 eV, which is an intrinsic property of the material. Decreasing the energy band would allow for the activity of TiO_2_ by UV light, which accounts for 5 % of the whole spectrum of sunlight. Therefore, broadening the responding spectral region of TiO_2_ is an effective method to increase the photocatalytic property of such materials [2, 3].

Electrospinning is a novel way to obtain one-dimensional inorganic nanofibers, such as TiO_2_, ZnO, Al_2_O_3_, SnO_2_, and BaTiO_3_. The electrospinning equipment consists of a high-voltage power supply, a spinneret, a syringe pump, and a collector. The precursor solution is jetted by the high-voltage power supply via the spinneret and the syringe pump, and nanofibers can then be obtained on the collector. By controlling the viscosity of the precursor, the high-voltage strength, and the distance between the spinneret and the collector, which could all affect the diameter and morphology of the nanofibers, we were able to obtain TiO_2_ polymers and amorphous TiO_2_ by annealing the nanofibers prepared via the electrospinning process [4, 5]. Vermiculite is a low cost and abundant layered silicate mineral raw material. It features a large surface area and specific absorption property. The nanofiber photocatalytic material used in this study was obtained by combing vermiculite and TiO_2_ nanofibers, which increases the volume surface area of the composites fibers and promotes the fibers’ ability to decompose chemical wastes materials [6, 7].

In this study, vermiculite and TiO_2_ composite nanofibers were synthesized by combining the sol–gel process with the electrospinning technique. The photocatalytic ability of the nanofiber composites was assessed by studying the degradation of methylene blue (MB) under irradiation with UV light. The results showed that the utilization of TiO_2_ composite nanofibers containing 2 wt.% of vermiculite resulted in a remarkable absorption and an enhanced degradation of MB.

## Methods

### Synthesis of the TiO_2_-Vermiculite Composite Nanofibers

The initial step in the synthesis of the TiO_2_-vermiculite composite nanofibers was the preparation of a precursor via the sol–gel process. To fabricate the precursor, 1.5 g of polyvinyl pyrrolidone (PVP, Mc = 1,300,000, Alfa Aesar), 20 ml of ethanol, 5 ml of acetic acid, and 5 ml of tetrabutyltitanate were mixed with different amounts of vermiculite powders and stirred for 2 h to ensure a homogeneous mixture. The obtained precursors were then transferred into the electrospinning instrument to fabricate the vermiculite/PVP/Ti(OCH(CH_3_)_2_)_4_ nanofibers. An electrical potential of 15 kV was applied between the nozzle and the collector in order to eject the sol–gel precursor to the collection board which was installed a distance of 15 cm to the nozzle. In order to obtain nanofibers with a homogenous size distribution, the propulsion speed was set to 2 ml/h. After a thermal treatment for 3 h at 550 °C, the vermiculite/PVP/Ti(OCH(CH_3_)_2_)_4_ fibers were transformed into TiO_2_-vermiculite composite nanofibers.

### Characterization

The vermiculite/PVP/Ti(OCH(CH_3_)_2_)_4_ fibers were characterized by the simultaneous application of thermogravimetry and differential scanning calorimetry (TG-DSC). The phase composition of the TiO_2_-vermiculite composite nanofibers was studied by D/max-rA X-ray diffraction (XRD, Rigaku Corporation, Japan, Cu Kα radiation, λ = 1.5406 Å). The microstructures and nanostructures of the nanofibers were characterized by scanning electron microscopy (SEM, JEOL JSM6700F, Japan) and transmission electron microscopy (TEM, 300 kV, FEI-tecnai-G^3^-F20, Philips, Netherlands). The nanofibers were ultrasonically dispersed in ethanol and then dropped onto carbon-coated copper grids prior to the TEM investigations. Diffuse reflectance ultraviolet–visible (UV–vis) absorption spectra were recorded using a Carry 5000 UV–vis-NIR spectrophotometer with an integrating sphere attachment.

The photocatalytic property of the nanofibers was evaluated as follow: the nanofiber samples were suspended into the MB solution (10 mg/L) and the solution was stirred for 30 min under exclusion of light. The adsorption equilibrium of the solution was reached when the methylene blue concentration remained in balance. Then, the solution was stirred by magnetic stirring and irradiated with UV light using a 500 W high-pressure mercury lamp performance in order to evaluate the photocatalytic property. A 3 ml of the pollutant solution was sampled every 20 min and centrifuged for the separation of the upper clear solution. The concentration of MB was analyzed using a UV–vis spectrophotometer (L5, INESA) at the characteristic wavelength of 664 nm.

## Results and Discussion

**Fig. 1:**
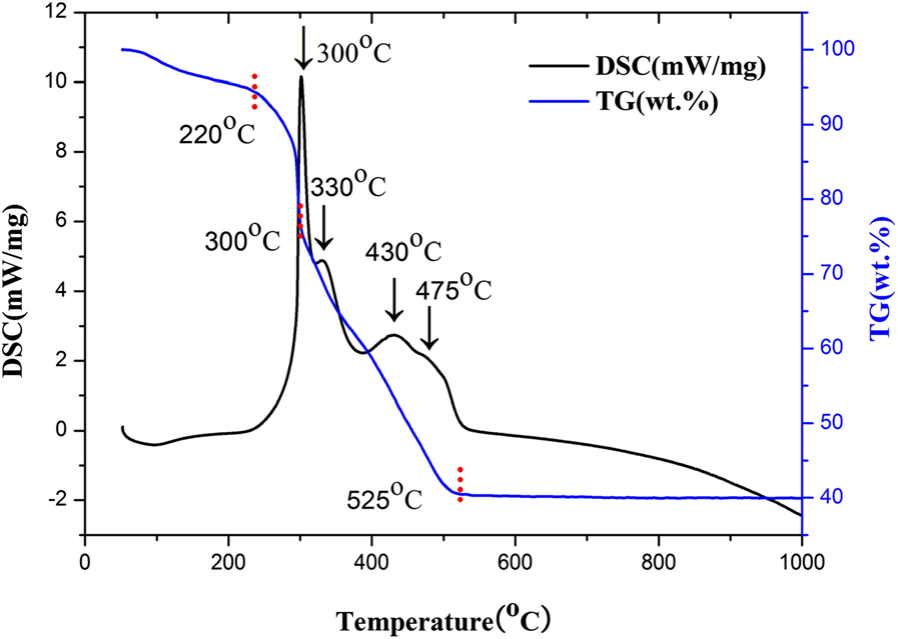
TG-DSC curve obtained for the PVP/Ti(OCH(CH_3_)_2_)_4_ composite nanofibers in air

The results of TG-DSC analyses of the PVP/Ti(OCH(CH_3_)_2_)_4_ composite nanofibers are shown in Fig. 1. The TG curves feature four distinct stages of weight loss: a weight loss of 5 % was observed when the temperature was increased from room temperature to 220 °C, a loss of 19 % occurred when the temperature was increased from 220 to 300 °C, another loss of 36 % occurred when the temperature was further increased from 300 to 525 °C, where no weight loss was detected for temperatures above 525 °C. The first weight loss was attributed to the evaporation of ethanol and water in the sample. The second weight loss, corresponding to the sharp peak in the DSC curve, was attributed to the decomposition of PVP side chain [8, 9]. Three exothermic peaks were observed during the third weight loss period in the temperature range from 300 to 430 °C, which were linked to the degradation of the PVP main chain and Ti(OCH(CH_3_)_2_)_4_. The formation of the anatase phase of TiO_2_ began at a temperature of 475 °C. The phase transformation from the anatase phase to the rutile phase was completed for temperatures above 525 °C [10, 11].

**Fig. 2:**
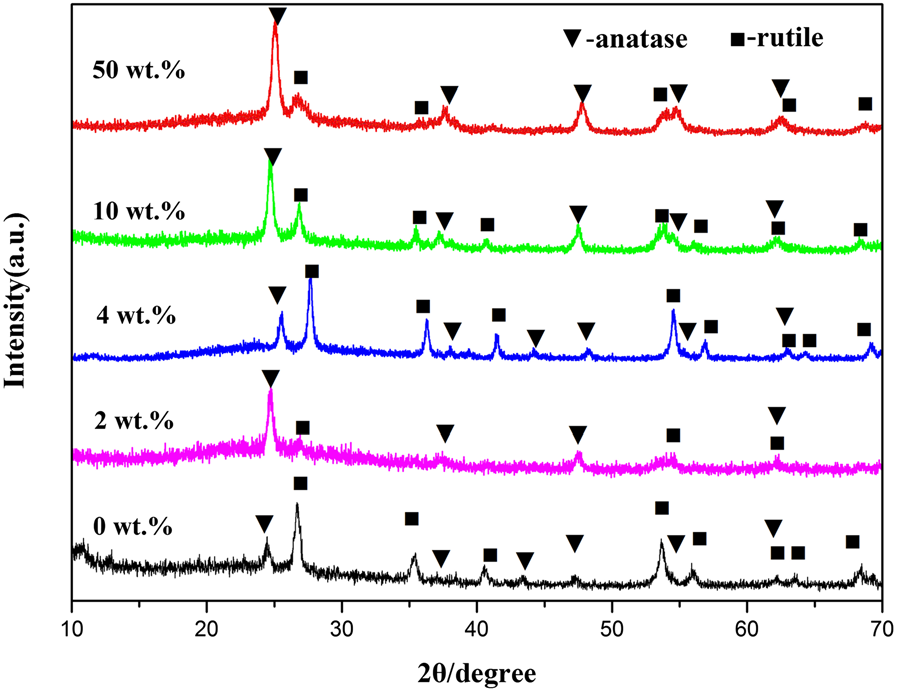
XRD patterns obtained for TiO_2_ nanofibers with a different vermiculite mass fraction(2 wt.%, 4 wt.% and 10 wt.%, 50 wt.%) and pure TiO_2_ nanofibers prepared at 550 °C for 3 h

Figure 2 shows the XRD spectra obtained for the TiO_2_-vermiculite composite nanofibers calcined at 550 °C for 3 h. The samples were mainly consist of anatase and rutile TiO_2_. The Anatase and rutile TiO_2_ are the main phases in pure TiO_2_ with more rutile TiO_2_ observed than anatase TiO_2_. After calcination of the composite nanofibers at temperatures of up to 550 °C, the anatase phase was the major phase in the pure TiO_2_, and only a small trace of the rutile phase remained. With the increase of the vermiculite mass fraction, the intensity of the peak of corresponding to the rulite phase increased compared to the corresponding peak observed for the pure TiO_2_ nanofibers. Interestingly, the anatase phase was only detected when at least 2 wt.% of vermiculite or more was added to the TiO_2_. Both the anatase and the rutile phase were found in the composite nanofibers after calcination, with the fraction of the anatase phase being higher than the fraction of the rutile phase. With the increase of the vermiculite mass fraction, the fraction of the rutile phase in the TiO_2_-vermiculite composite nanofibers significantly increased. Evidently, the addition of vermiculite had an effect on the phase composition of the TiO_2_ nanofibers.

**Fig. 3:**
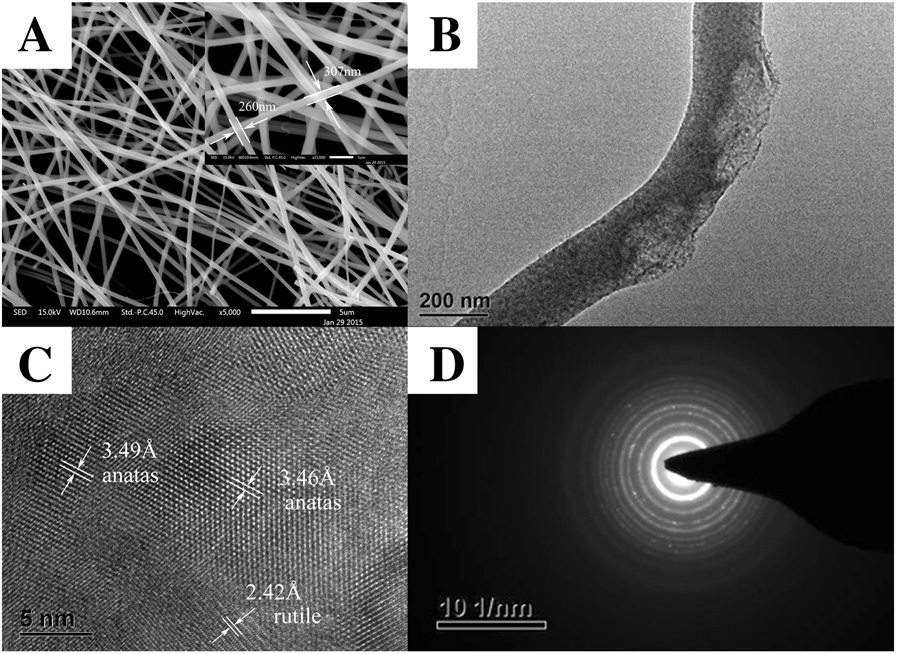
**a**, **b** Microtopography of the TiO_2_-vermiculite composite nanofibers with 2 wt.% vermiculite, **c** HRTEM micrograph of the TiO_2_-vermiculite composite nanofibers with 2 wt.% vermiculite, **d** electron diffraction pattern obtained for the composite nanofibers

Figure 3a shows SEM micrographs of the TiO_2_-vermiculite composite nanofibers with 2 wt.% vermiculite. It can be clearly seen that the samples contained smooth and continuous nanofibers with a diameter between 250 and 300 nm. In Fig. 3b, vermiculite particle was found among the TiO_2_ nanofibers. Such particles can adsorb the pollutant and increase the concentration of the reactants thereby promoting the degradation reaction. However, when the number of vermiculite particles in the composites increases, the available amount of the TiO_2_ catalyst decreases and the photocatalytic property is reduced.

Both the anatase and the rutile phase were found in the composite nanofibers exposed to the thermal treatment at 550 °C (Fig. 3c). In addition, the electron diffraction pattern obtained for the TiO_2_-vermiculite composite nanofibers also revealed that the nanofibers are polycrystalline. As demonstrated in literature, the coexistence of the anatase and the rutile phase may enhance the photocatalytic property [12, 13]. Therefore, for a suitable TiO_2_ nanofibers to vermiculite particle ratio (here, 2 wt.% of vermiculite particles), the TiO_2_-vermiculite composite nanofibers show an optimized photocatalytic property.

**Fig. 4:**
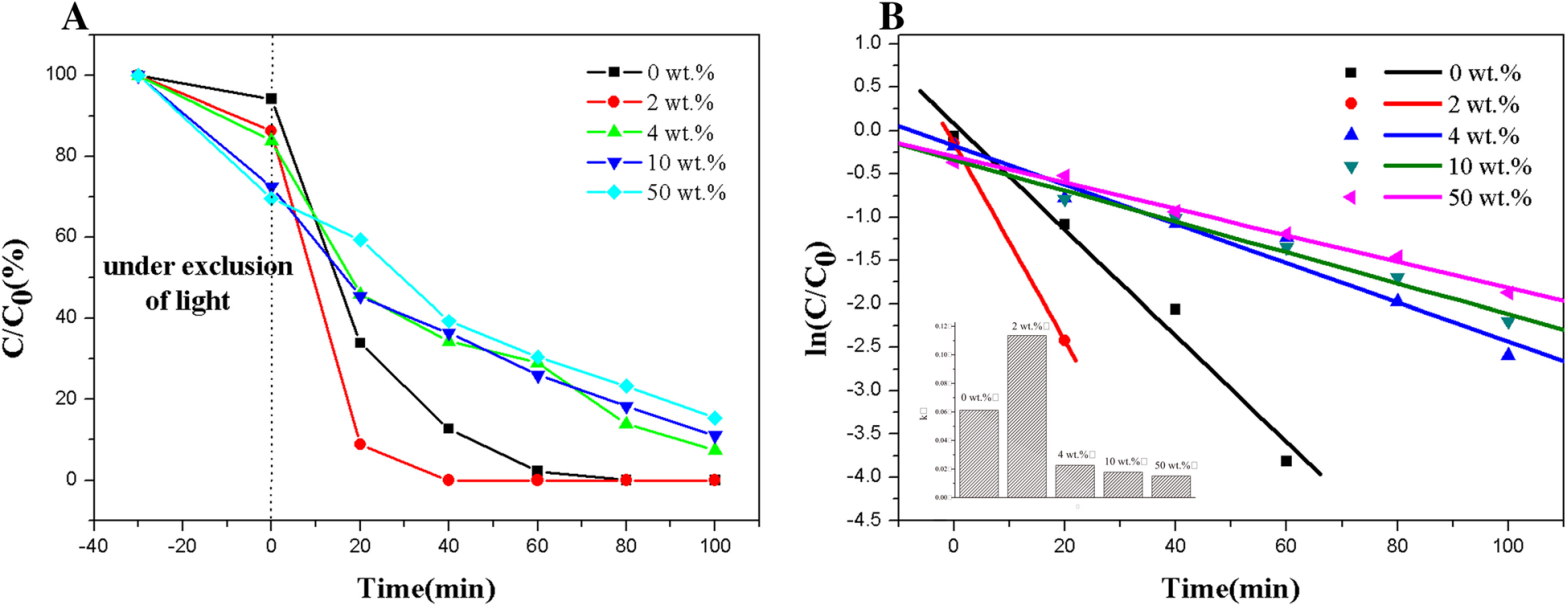
Comparison of the photocatalytic degradation of methylene blue by TiO_2_ nanofibers with different vermiculite mass fraction prepared at 550 °C for 3 h (**a** degradation curve, **b** kinetic curve)

Figure 4a shows the photocatalytic degradation behavior of TiO_2_-vermiculite composite nanofibers for the degradation of MBin a solution with an MB concentration of 10 mg/L. As shown in Fig. 4a, the initial MB concentration is lower than 100 % after stirring for 30 min under exclusion of light. Furthermore, the MB concentration is lower when the amount of vermiculite in the nanofibers is higher. In this adsorption process, the vermiculite acts as an adsorbing material. Under irradiation with UV light, the composite nanofibers degrade the MB in the solution, which is a photodegradation process. Therefore, in the experiments, an adsorption-photodegradation process occurred. As a result, the samples containing 2 wt.% vermiculite showed the best adsorption and photocatalytic behavior. Compared with the pure TiO_2_ nanofibers, for the TiO_2_ composite nanofibers containing 2 wt.% vermiculite, the reaction equilibrium is reached earlier.

The photodegradation rate is an important factor for the evaluation of photocatalytic materials. Figure 4b compares the degradation rate of the samples. In Fig. 4b, the slope of the line corresponds to the reaction rate of the photodegradation process. The degradation rate of the TiO_2_ nanofibers with 2 wt.% vermiculite nanoparticles is the highest among all samples. This can be explained as follows: The degradation process is caused by the TiO_2_. With an increasing amount of vermiculite and a decreasing amount of the catalyst (TiO_2_), the absorbability is enhanced, whereas the catalytic ability is reduced at the same time. Therefore, the TiO_2_ nanofibers with 2 wt.% vermiculite nanoparticles show stronger capabilities (including adsorption and photocatalysis) to treat chemical pollutions.

**Fig. 5:**
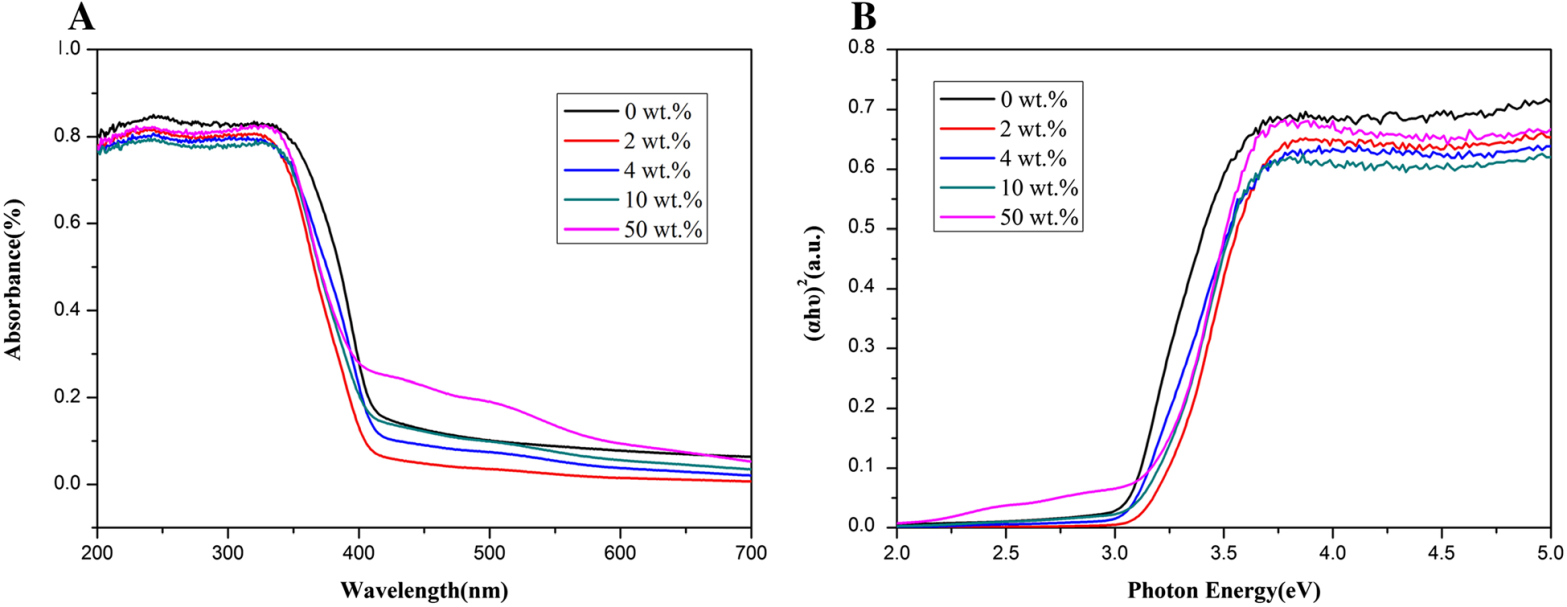
**a** Comparison of the UV–vis absorption spectra obtained for TiO_2_ nanofibers with different vermiculite mass fractions calcined in air at 550 °C for 3 h and **b** (*αhυ*)^2^ plotted as a function of the photon energy, *E*_*g*_, for the different TiO_2_-vermiculite composite nanofibers

The UV–vis absorption spectra of the TiO_2_ composite nanofibers with different vermiculite mass fraction are compared in Fig. 5a, displaying the absorption spectra for each sample for an excitation wavelength of approximately 400 nm (3.10 eV). The band gap energy (*E*_*g*_) for each sample was obtained by plugging the absorption data into the direct transition equation:

$$ \alpha h\upsilon ={E}_D{\left(h\upsilon -{E}_g\right)}^{1/2} $$

- *α* - optical absorption coefficient;
- *υ* - photon energy;
- *E*_*g*_ - direct band gap;
- *h and E*_*D*_ - a constant.

The tangent to the curves (Fig. 5b) at the point of intersection with the x-axis corresponds to the direct band gap energy (*E*_*g*_) of the samples, which are 3.05, 3.14, 3.08, 3.17, and 3.18 eV for a vermiculite mass fraction of 0, 2, 4, 10, and 50 wt.%, respectively [Additional files 1, 2, 3, 4 and 5]. The band gap energy increases with the amount of vermiculite in the nanofibers (Fig. 5b). The band gap energy of pure TiO_2_ nanofibers is 3.05 eV due to the band alignment of the rutile and anatase TiO_2_ phase. Moreover, the addition of a small amount of vermiculite to the TiO_2_ nanofibers significantly changed the band gap energy. However, adding a larger amount of vermiculite to the nanofibers had negative effect on the band gap. In this paper, the composite nanofibers with 2 wt.% vermiculite showed the most suitable direct band gap and the highest photocatalytic activity.

## Conclusions

TiO_2_-vermiculite nanofiber composites were synthesized by combining a sol–gel process with the electrospinning technique. The TiO_2_-vermiculite nanofiber composites were obtained after thermal treatment at 550 °C for 3 h. The results of the phase composition analysis indicate that the main phase of the prepared samples was the anatase phase, and a small amount of the rutile phase could also be detected. The analysis of the structure of the composite nanofibers revealed a smooth surface and diameter of approximately 300 nm. The photodegradation of methylene blue by the as-prepared TiO_2_-vermiculite composite nanofibers was performed under irradiation lights. In this study, the addition of the mineral powder to the TiO_2_ nanofibers was demonstrated to enhance the adsorption-photocatalytic performance of the photocatalytic material.

## Additional files

Additional file 1:
***(αhυ)*** ^**2**^ **plotted as a function of the photon energy,** ***Eg*** **, for pure TiO**_**2**_ **nanofibers.**
Additional file 2:
***(αhυ)*** ^**2**^ **plotted as a function of the photon energy,** ***Eg*** **, for composite nanofibers with 2 wt.% vermiculite.**
Additional file 3:
***(αhυ)*** ^**2**^ **plotted as a function of the photon energy,** ***Eg*** **, for composite nanofibers with 4 wt.% vermiculite.**
Additional file 4:
***(αhυ)*** ^**2**^ **plotted as a function of the photon energy,** ***Eg*** **, for composite nanofibers with 10 wt.% vermiculite.**
Additional file 5:
***(αhυ)*** ^**2**^ **plotted as a function of the photon energy,** ***Eg*** **, for composite nanofibers with 50 wt.% vermiculite.**

## References

[1] Woan K, Pyrgiotakis G, Sigmund W (2009). Photocatalytic carbon-nanotube-TiO_2_ composites. Adv Mater.

[2] Ni M, Leung MKH, Leung DYC (2007). A review and recent developments in photocatalytic water-splitting using TiO_2_ for hydrogen production. Renew Sust Energ Rev.

[3] Fujishima A, Zhang X, Tryk DA (2008). TiO_2_ photocatalysis and related surface phenomena. Surf Sci Rep.

[4] Li D, Xia Y (2003). Fabrication of titania nanofibers by electrospinning. Nano Lett.

[5] Li H, Zhang W, Li B (2010). Diameter‐dependent photocatalytic activity of electrospun TiO_2_ nanofiber. J Am Ceram Soc.

[6] Chen Q, Wu P, Dang Z (2010). Iron pillared vermiculite as a heterogeneous photo-Fenton catalyst for photocatalytic degradation of azo dye reactive brilliant orange X-GN. Sep Purif Technol.

[7] Wang L, Wang X, Cui S (2013). TiO_2_ supported on silica nanolayers derived from vermiculite for efficient photocatalysis. Catal Today.

[8] Daβler A, Feltz A, Jung J (1988). Characterization of rutile and anatase powders by thermal analysis. J Therm Anal.

[9] Azhari SJ, Diab MA (1998). Thermal degradation and stability of poly (4-vinylpyridine) homopolymer and copolymers of 4-vinylpyridine with methyl acrylate. Polym Degrad Stab.

[10] Nuansing W, Ninmuang S, Jarernboon W (2006). Structural characterization and morphology of electrospun TiO_2_ nanofibers. Mater Sci Eng B.

[11] Li JY, Dai H, Li Q (2006). Lanthanum zirconate nanofibers with high sintering-resistance. Mater Sci Eng B.

[12] Scanlon DO, Dunnill CW, Buckeridge J (2013). Band alignment of rutile and anatase TiO_2_. Nat Mater.

[13] Zhang J, Xu Q, Feng Z (2008). Importance of the relationship between surface phases and photocatalytic activity of TiO_2_. Angew Chem Int Ed.

