# Supplementary figures and images for: Photocatalytic Property of TiO2-Vermiculite Composite Nanofibers via Electrospinning
Source: Nanoscale Res Lett. 2015 Jul 1;10:276. doi: 10.1186/s11671-015-0977-1 (PMC4486496; doi:10.1186/s11671-015-0977-1)

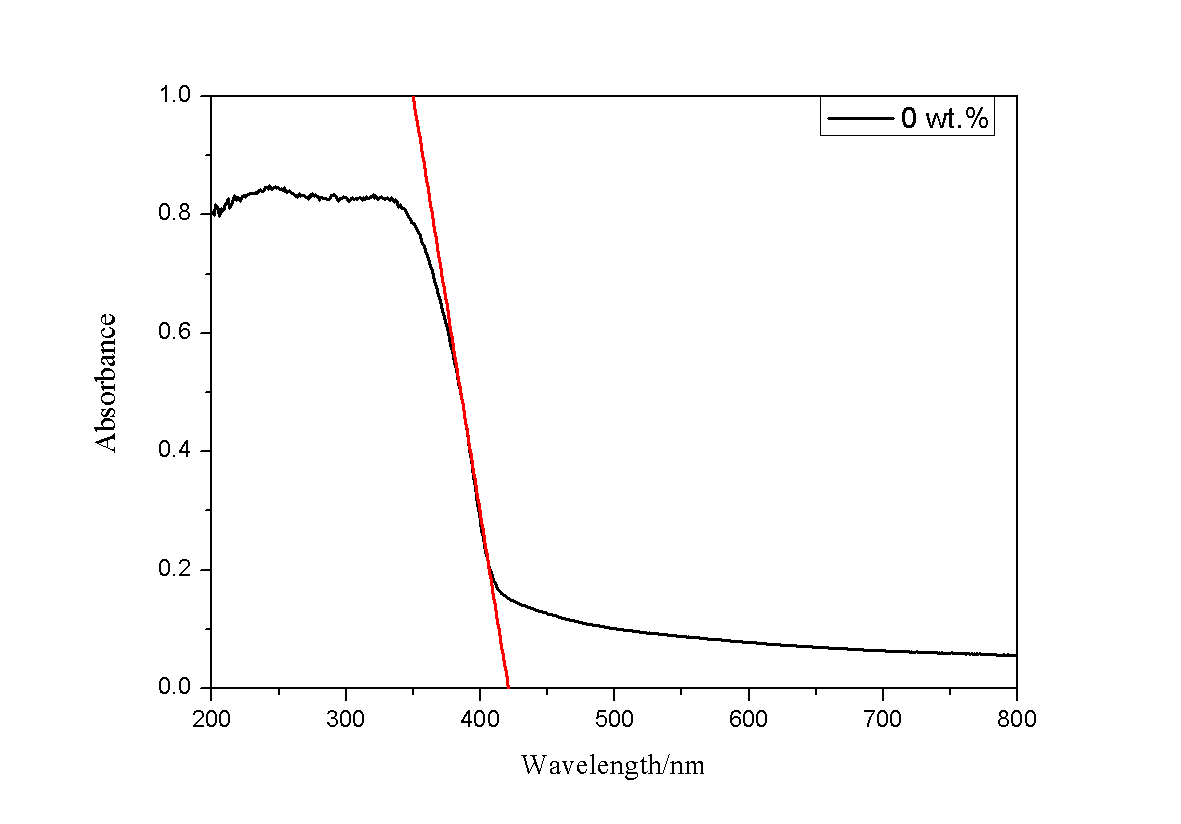

Supplement: Additional file 1: — (αhυ) 2 plotted as a function of the photon energy, Eg , for pure TiO 2 nanofibers. [file 11671_2015_977_MOESM1_ESM.png]

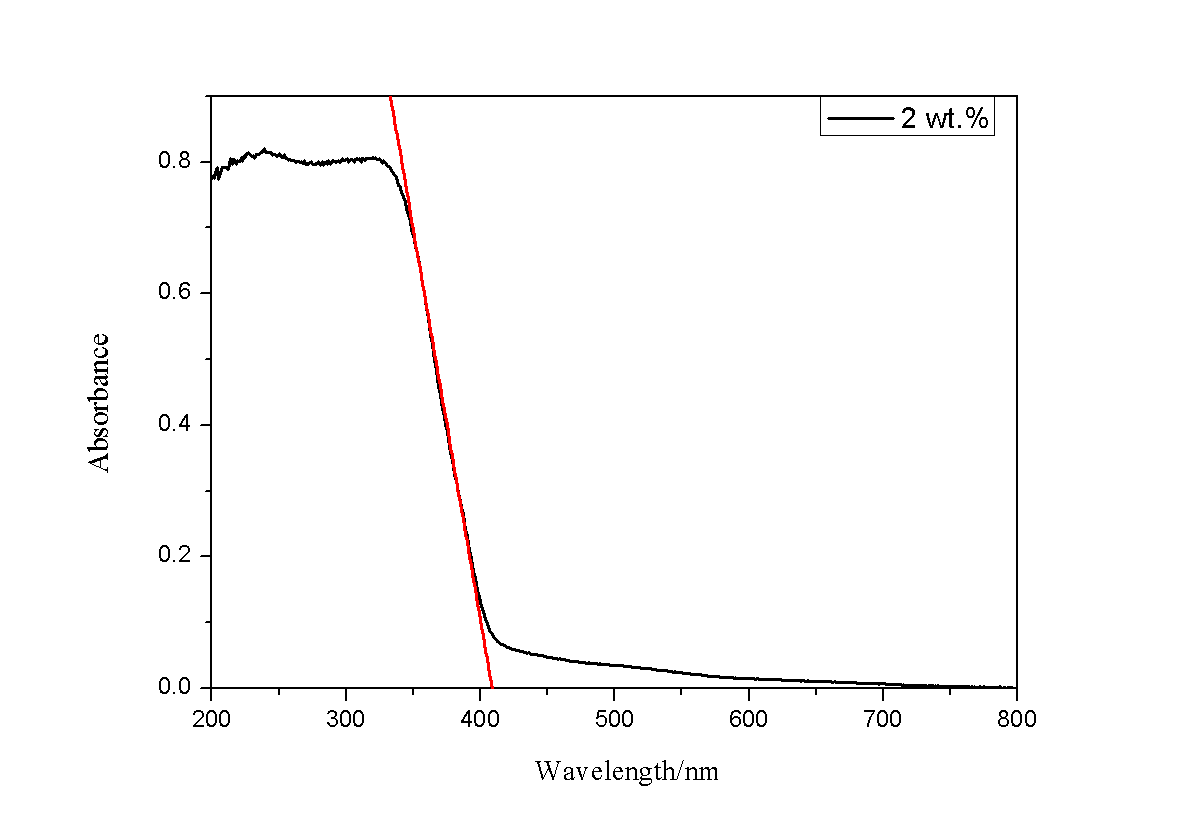

Supplement: Additional file 2: — (αhυ) 2 plotted as a function of the photon energy, Eg , for composite nanofibers with 2 wt.% vermiculite. [file 11671_2015_977_MOESM2_ESM.png]

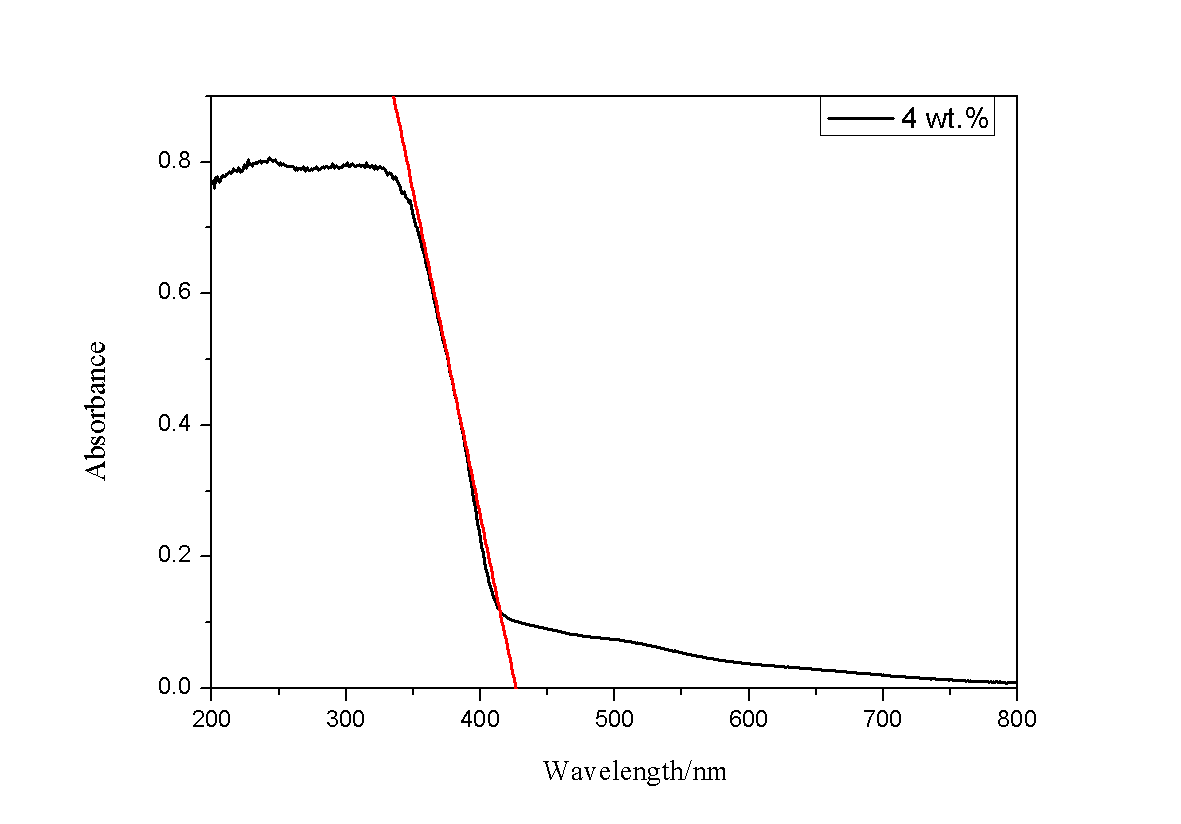

Supplement: Additional file 3: — (αhυ) 2 plotted as a function of the photon energy, Eg , for composite nanofibers with 4 wt.% vermiculite. [file 11671_2015_977_MOESM3_ESM.png]

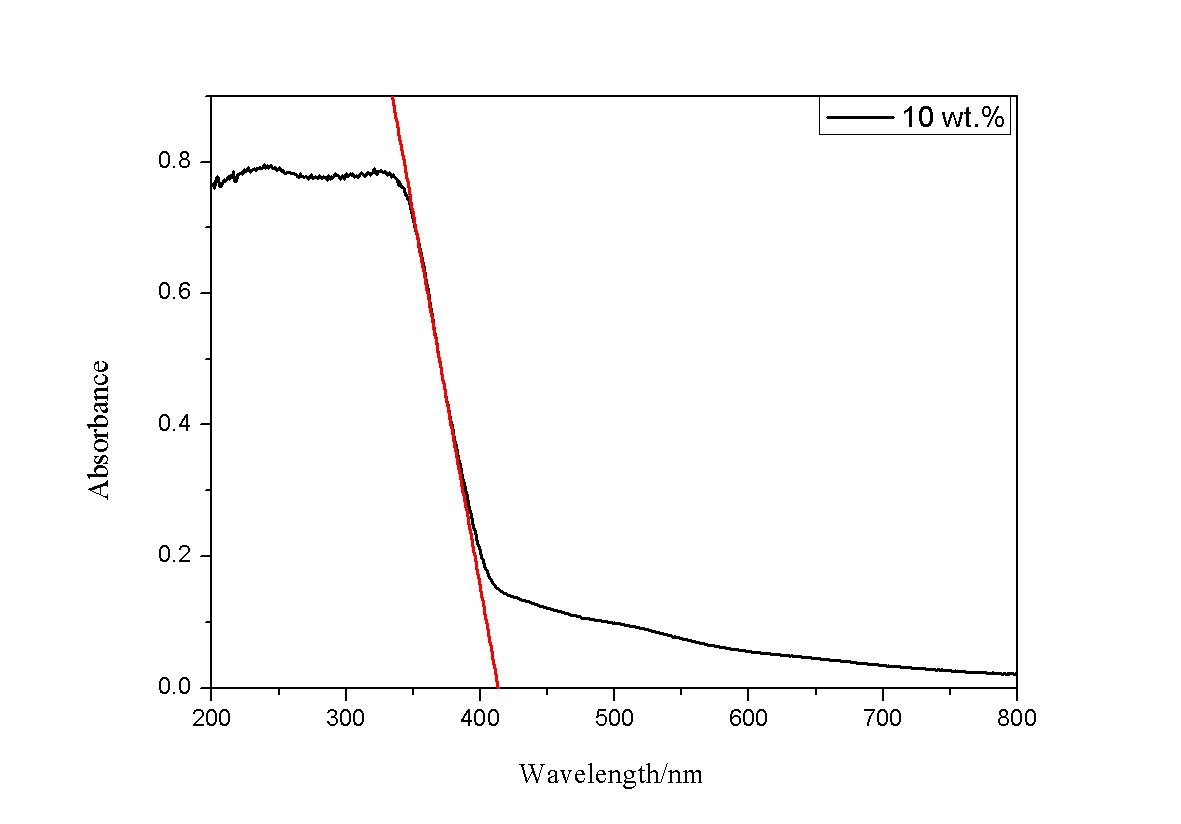

Supplement: Additional file 4: — (αhυ) 2 plotted as a function of the photon energy, Eg , for composite nanofibers with 10 wt.% vermiculite. [file 11671_2015_977_MOESM4_ESM.png]

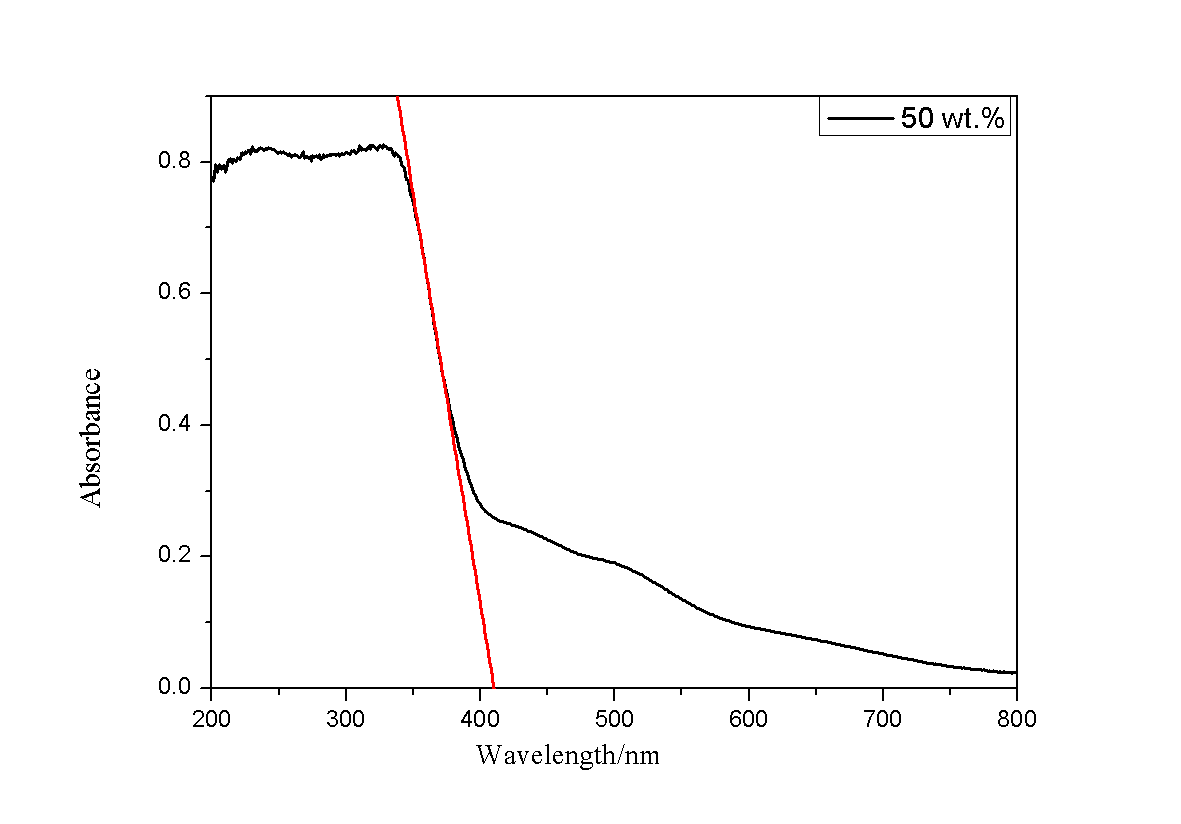

Supplement: Additional file 5: — (αhυ) 2 plotted as a function of the photon energy, Eg , for composite nanofibers with 50 wt.% vermiculite. [file 11671_2015_977_MOESM5_ESM.png]
